# Quality of cause-of-death reporting using ICD-10 drowning codes: a descriptive study of 69 countries

**DOI:** 10.1186/1471-2288-10-30

**Published:** 2010-04-08

**Authors:** Tsung-Hsueh Lu, Philippe Lunetta, Sue Walker

**Affiliations:** 1 NCKU Research Center of Health Data and Institute of Public Health, College of Medicine, National Cheng Kung University, No. 1, Dah Hsueh Road, Tainan, Taiwan; 2 Hjelt Institute, Department of Forensic Medicine, University of Helsinki, and National Institute for Health and Welfare, Injury Prevention Unit, 00300 Helsinki, Finland; 3 National Centre for Health Information Research and Training, School of Public Health, Queensland University of Technology, Victoria Park Road, Kelvin Grove, Australia

## Abstract

**Background:**

The systematic collection of high-quality mortality data is a prerequisite in designing relevant drowning prevention programmes. This descriptive study aimed to assess the quality (i.e., level of specificity) of cause-of-death reporting using ICD-10 drowning codes across 69 countries.

**Methods:**

World Health Organization (WHO) mortality data were extracted for analysis. The proportion of unintentional drowning deaths coded as unspecified at the 3-character level (ICD-10 code W74) and for which the place of occurrence was unspecified at the 4^th ^character (.9) were calculated for each country as indicators of the quality of cause-of-death reporting.

**Results:**

In 32 of the 69 countries studied, the percentage of cases of unintentional drowning coded as unspecified at the 3-character level exceeded 50%, and in 19 countries, this percentage exceeded 80%; in contrast, the percentage was lower than 10% in only 10 countries. In 21 of the 56 countries that report 4-character codes, the percentage of unintentional drowning deaths for which the place of occurrence was unspecified at the 4^th ^character exceeded 50%, and in 15 countries, exceeded 90%; in only 14 countries was this percentage lower than 10%.

**Conclusion:**

Despite the introduction of more specific subcategories for drowning in the ICD-10, many countries were found to be failing to report sufficiently specific codes in drowning mortality data submitted to the WHO.

## Background

Drowning is an important but neglected global health issue [1-4]. The systematic collection of high-quality mortality data about the environmental events and circumstances leading to a case of drowning is a prerequisite to designing relevant drowning prevention programmes [5,6]. Almost all countries collect, classify and tabulate cause-of-death data according to the same standard procedure (i.e., using the *International Statistical Classification of Diseases and Related Health Problems *(ICD) published by the World Health Organization (WHO)) [7]. The update from the *Ninth Revision *(ICD-9) to the *Tenth Revision *(ICD-10) entailed significant changes in the classification of unintentional drowning [8]: the ICD-9 emphasised details regarding the nature of the recreational activity undertaken at the time of the event; in contrast, the ICD-10 highlights details about the nature of the body of water (e.g., bathtub, swimming pool or natural water) and the mechanism of drowning (e.g., while in water *versus *following a fall into water) (Table 1).

**Table 1:** Sub-categories of ICD-9 and ICD-10 codes for unintentional drowning (online ICD-10 codes can be accessed at http://apps.who.int/classifications/apps/icd/icd10online).

ICD-9 code		ICD-10 code	
E910	Accidental drowning and submersion	W65--W74	Drowning and submersion
E910.0	while water-skiing	W65	while in bathtub
E910.1	while engaged in other sport or recreational activity with diving equipment	W66	following fall into bathtub
E910.2	while engaged in other sport or recreational activity without diving equipment	W67	while in swimming pool
E910.3	while swimming or diving for purposes other than recreation or sport	W68	following fall into swimming pool
E910.4	in bathtub	W69	while in natural water
E910.8	Other	W70	following fall into natural water
E910.9	unspecified place of occurrence code	W73	other specified
		W74	unspecified

In the ICD-10, the following fourth-character sub-divisions serve to identify the place of occurrence of the external cause where relevant:

.0 Home

.1 Residential institution

.2 School, other institution, or public administrative area

.3 Sports and athletics area

.4 Street or highway

.5 Trade and service area

.6 Industrial or construction area

.8 Other specified place

.9 Unspecified place

Despite the innovative expansion of the classification scheme in the ICD-10 for unintentional drowning, little is known about the current quality of cause-of-death reporting using ICD-10 codes in most countries. As noted in the *World Report on Child Injury*, even in relatively advanced countries, information regarding the place in which drowning occurs is poorly documented on death certificates [[9], p.61]. A recent study also indicates that of the 52 countries in the WHO European region, only 23 use 4th-character subdivisions and only 3 countries had high-quality data on the place of occurrence of injuries [10]. This present study aimed to assess the quality (i.e., level of specificity) of cause-of-death reporting using ICD-10 drowning codes across countries.

## Methods

Data were extracted from the WHO mortality database for analysis [11]. As of August 2009, 146 countries submitted mortality data to the WHO, of which 17 used only 3-character ICD-10 codes and 84 used 4-character ICD-10 codes. The ICD-10 codes for unintentional drowning are W65-W74 (Table 1). We excluded suicide by drowning (ICD-10 code X71), homicide by drowning (ICD-10 code X92), and cases of drowning of undetermined intent (ICD-10 code Y21), because no specific sub-categories exist for these codes in the ICD-10.

To ensure statistical stability in the calculation of percentages, we included only those countries with more than 20 reported unintentional drowning deaths; thus, a total of 69 countries were included in the final analysis. To illustrate the magnitude of the mortality rates of unintentional drowning for each country, we first computed the age-adjusted drowning death rate (deaths per 100 000 people) for each country using the WHO standard population structure. The age groups used for computing age-adjusted death rates were 0-14, 15-24, 25-44, 45-64 and 65+ years of age. Of the 69 countries included in the analysis, only 43 countries had population data from which the death rate could be calculated.

We then calculated two indicators of the quality (i.e., level of specificity) of cause-of-death reporting using ICD-10 codes for cases of unintentional drowning. The first indicator was the proportion of unintentional drowning deaths (ICD-10 codes W65-W74) coded as unspecified at the 3-character level (ICD-10 code W74) (Table 1); the second was the proportion of unintentional drowning deaths in which the place of occurrence was unspecified at the 4^th ^character (.9) (Table 1). In other words, the higher the percentage of cases in which an unspecified code is reported, the poorer the quality of cause-of-death reporting.

## Results

Of the 43 countries with population data available for the calculation of age-adjusted death rates, five countries had a drowning death rate higher than 5 per 100 000: 9.8 in Lithuania, 9.6 in Latvia, 7.1 in Thailand, 6.9 in the Republic of Moldova, and 5.4 in Kyrgyzstan (Table 2). In 32 of the 69 countries studied in total, the percentage of cases of unintentional drowning coded as unspecified at the 3-character level exceeded 50%, and in 19 countries, this percentage exceeded 80%; in contrast, this proportion was lower than 10% in only 10 countries. In 21 of the 56 countries that report 4-character codes, the proportion of unintentional drowning deaths in which the place of occurrence was unspecified at the fourth character exceeded 50%, and in 15 countries, the percentage exceeded 90%; in only 14 countries was this percentage lower than 10%. We found a large discrepancy between countries with regard to the unspecified codes at the 3-character level and the 4^th ^character code; for example 99% vs. 0% respectively in El Salvador, and, in contrast, 0% versus 100% in New Zealand.

**Table 2:** Number and age-adjusted death rate (per 100 000 people) of unintentional drowning deaths and the proportion of drowning deaths coded as 'unspecified' in each country, ranked by the percentage of 'unspecified' cases at the 3-character level according to the WHO mortality database, August 2009.

Country	The latest available year	No. of deaths from unintentional drowning	Age- adjusted drowning death rate	% of drowning deaths classified as unspecified at the 3-character level	% of drowning deaths classified as unspecified at the 4th character
Suriname	2005	34	NA	100	94
Kuwait	2002	20	1.7	100	90
Guatemala	2006	87	NA	100	61
Thailand	2002	4218	7.1	100	100
Peru	2000	656	NA	100	96
El Salvador	2006	238	NA	99	0
Guyana	2005	90	NA	99	100
Israel	2005	48	0.7	98	100
Belize	2001	41	NA	98	100
Mauritius	2007	38	3.1	97	100
Uruguay	2004	71	NA	97	44
South Africa	2005	147	NA	96	NA
Costa Rica	2006	132	NA	94	6
Taiwan	2007	486	1.9	89	NA
Georgia	2001	45	0.9	89	NA
Chile	2005	488	NA	89	1
Bahamas	2002	42	NA	86	52
Serbia and Montenegro, Former	2002	94	1.2	84	11
France	2006	1008	1.3	82	53
Réunion	2005	20	NA	80	55
Italy	2006	378	0.6	75	49
Paraguay	2004	99	NA	69	21
Spain	2005	494	1.0	66	47
Haiti	2003	20		65	45
Norway	2006	65	1.1	63	100
Argentina	2005	563	NA	60	17
Republic of Moldova	2007	261	6.9	60	0
Kyrgyzstan	2006	270	5.4	57	23
Netherlands	2007	76	0.4	57	3
Egypt	2000	1591	NA	54	NA
Uzbekistan	2005	1042	3.9	52	NA
Azerbaijan	2007	62	0.8	52	NA
Romania	2007	992	4.4	50	18
Serbia	2007	115	1.4	46	NA
Mexico	2006	2310	NA	46	21
Colombia	2005	1019	NA	44	16
Ecuador	2006	521	NA	44	21
Czech Republic	2007	182	1.5	44	31
Denmark	2006	46	0.7	43	100
Germany	2006	418	0.4	43	31
Brazil	2005	6171	NA	42	26
Republic of Korea	2006	757	1.5	40	NA
Venezuela	2005	590	NA	38	17
Sweden	2006	106	0.9	38	100
Nicaragua	2005	196	NA	37	97
Belgium	1999	54	0.5	37	NA
Poland	2006	1031	2.5	34	52
Canada	2004	251	0.8	32	100
Croatia	2006	78	1.3	32	36
Austria	2007	75	0.8	27	21
United Kingdom	2007	224	0.4	24	2
United States of America	2005	3582	1.2	23	10
Puerto Rico	2005	37	NA	19	0
Australia	2004	198	1.0	19	12
Estonia	2005	59	4.1	17	NA
Hungary	2005	192	1.7	14	2
Trinidad and Tobago	2002	44	3.4	14	11
Dominican Republic	2004	29	NA	10	100
Ireland	2007	51	1.1	10	4
Japan	2007	5966	2.5	9	7
Slovenia	2007	28	1.1	7	NA
Hong Kong SAR	2007	43	NA	7	0
Slovakia	2005	138	2.4	7	NA
Lithuania	2007	381	9.8	6	1
Panama	2006	120	NA	5	9
Cuba	2006	253	NA	1	2
Latvia	2007	237	9.6	1	NA
Finland	2007	143	2.1	1	100
New Zealand	2005	56	1.4	0	100

## Discussion

Using the percentage of cases coded as unspecified as an indicator of the quality of cause-of-death reporting for unintentional drowning deaths using ICD-10 codes, our findings indicate that in one in seven of the countries studied the quality of cause-of-death reporting was less than acceptable. Factors associated with the coding of unintentional drowning deaths as unspecified may result from different factors, such as a lack of specific information regarding the circumstances that led to the drowning, the inadequate collection of primary data owing to insufficient police and medico-legal investigation, and incompleteness or errors during the death certification and coding process. In addition, countries may focus on different aspects of drowning for their prevention programmes and therefore require differing levels of specificity in the ICD-10 codes. For example, El Salvador may require more details about the place of occurrence of the drowning at the 4^th ^character code level and place less emphasis on the body of water, whereas in New Zealand the focus may be more on coding information about the body of water involved and less on the place of occurrence. Additionally, the use of a national modification of the ICD-10 in New Zealand may have had an effect on reporting of place of occurrence, as the modification utilizes different codes compared with the international version of the ICD-10.

An international comparison study indicated that the main reason for which injury-related deaths are coded as unspecified is that medical certifiers (including medical examiners and coroners) fail to report sufficiently detailed information on the death certificates to allow coders to assign specific codes [12]. Another study also indicated that despite the legal requirement that all unnatural deaths be subjected to forensic investigation by a physician in Thailand, the cause of death is usually described in terms of symptoms rather than attributed to a specific underlying cause, because physicians are reluctant to provide specifics and risk involvement in legal proceedings [13]. Parish also suggests that a lack of standardised methods and inadequate training for certifiers, medical examiners and coroners in addition to a lack of adequate resources for conducting investigations of deaths results in variations in the quality of mortality data for injury surveillance [14]. More efforts should focus on training medical certifiers to report specific information relevant to injury prevention on death certificates.

The circumstances and environments that result in drowning deaths differ between countries according to the reported ICD codes. The differentiation of mechanisms of drowning deaths into 'while in water' from 'following a fall into water' is one of the important changes in the ICD-10. The two circumstances have different implications for injury prevention. For instance, drowning while in water would mandate the promotion of personal flotation devices and ensuring adequate supervision, whereas drowning following a fall into water would underscore the value of effective barriers. A percentage of drowning deaths coded as unspecified that was high and skewed to one particular mechanism of drowning would bias comparisons of mortality between the two mechanisms. We recommend caution in interpreting the possible effects of unspecified coding on the comparison of international drowning mortality data by sub-category.

One of the limitations of this study was our use of secondary mortality data from the WHO, which lacks metadata about how each country collects information concerning circumstances resulting in and the mechanisms of unintentional drowning as well as the process of death certification and coding. The second limitation was that many countries do not yet use the ICD-10, and some countries had no available population data; having such data available would have contributed to a more complete picture of the global status. The third limitation was the difficulty in defining 'high quality' according to the percentage of cases coded as unspecified; in this study, we used a cut-off of 10%. There is no internationally recognized standard to calculate the quality of coded data. This study is simply an initial assessment of the global status of the provision of specific information in cases of death due to unintentional drowning, and further studies are needed to explore the exact reasons for the high number of cases of unintentional drowning coded as unspecified in various countries.

## Conclusion

In conclusion, despite the introduction of more specific subcategories for drowning in the ICD-10, which would provide better information for the design of prevention programmes, the findings of this study illustrate that many countries fail to report sufficiently specific codes in drowning mortality data submitted to the WHO.

## Competing interests

The authors declare that they have no competing interests.

## Authors' contributions

THL initiated this study and conducted the primary data analyses. THL, PL and SW participated equally in the interpretation of the results and critically commented upon and drafted the manuscript. All the authors have read and approved the final version of the manuscript.

## Pre-publication history

The pre-publication history for this paper can be accessed here:

http://www.biomedcentral.com/1471-2288/10/30/prepub
